# Recent Advances in Mesoporous Materials and Their Biomedical Applications

**DOI:** 10.3390/ijms232415636

**Published:** 2022-12-09

**Authors:** Juan Antonio Cecilia, Ramón Moreno-Tost

**Affiliations:** Departamento de Química Inorgánica, Cristalografía y Mineralogía, Universidad de Málaga (Unidad Asociada al ICP-CSIC), 29071 Málaga, Spain

Since the beginning of civilization, porous materials have been used for medical purposes. Some studies have reported that the first uses of applications of porous materials were carried out by the Ancient Egyptian or Western Africans, where porous charcoal or clay minerals were used as antidiarrheal medicine [1,2]. The use of charcoal continued to be used for medical purposes throughout history. Indeed, the Hindi civilization used charcoal for the purification of H_2_O [3]. More recently, the British Empire added charcoal to water barrels to increase the durability of drinking water [4]. Nowadays, charcoal is used as animal feed because it helps in the health and growth of the animals [5]. Regarding clay minerals, the use of kaolinite or montmorillonite is actually employed in some medicine such as Kaopectate^®^ due to its good antidiarrheal applications [2,6].

During the 18th and 19th centuries, scientific development promoted adsorption systems and more sophisticated applications. Thus, there is evidence of studies where adsorbents were used in purification processes of the stomach in the face of poisoning [7] or where porous natural materials were used in the face of ingestion of As-species [8]. The use of these traditional adsorbents also had military purposes since charcoal filters were used together with natural fibers in gas protection masks in the First World War [9].

In the last century, other alternative adsorbents to clay minerals and carbons have emerged, and new materials with greater porosity and a controlled pore diameter have been designed.

Although natural zeolites have been known since the 19th century, the study of synthetic zeolites began in the 1940s for use in adsorption and catalytic processes [10]. In the same way, SiO_2_ aerogels were developed in the 1930s [11]; however, one of the main advances in the design of adsorbents for biomedical purposes came from the synthesis of silica with its high specific surface area as well as controlled morphology and diameter [12,13]. These solids have found uses in many different fields of science in the last decades, from catalysis or adsorption to biomedical applications. One of the most significant breakthroughs is related to carrying a specific drug to its target and releasing it on demand upon stimulation. The controlled and sustained release of drug molecules from the ordered mesoporous structure can reduce the total dose, which can reduce side effects caused by the over-use of the drug and increase the efficiency of the drug’s action by increasing its local concentration. Considering these premises, the contributions of this Special Issue highlight some advances in the design and synthesis of mesoporous materials and their biomedical applications.

Cordeiro et al., designed and developed targeted mesoporous silica nanoparticles with the capability to deliver an anticancer drug specifically and efficiently to hepatocellular carcinoma (HCC) cells [14]. For that, mesoporous silica nanoparticles were functionalized with the targeting ligand triantennary N-acetylgalactosamine (GalNAc) cluster to promote a redox response, which exhibits high affinity to asialoglycoprotein receptors overexpressed in HCC cells and loaded with an anthracycline drug as epirubicin. The synthesized nanocarrier shows excellent physicochemical properties for drug delivery, high drug loading capacity, high biocompatibility, and a strong ability to target HCC cells, so this nanocarrier has a good biopharmaceutical potential as a targeted drug carrier for therapeutic applications in liver diseases [14].

Solarska-Sciuk et al., elucidate the mechanism underlying the cellular response to stress, which is induced by the exposure of cells to both biogenic and pyrogenic silica nanoparticles, which may lead to their death [15]. The obtained results pointed out the biological effects of mesoporous silica nanoparticles extracted from *Urtica dioica* L. and pyrogenic material, indicating that these silica nanoparticles have a clear impact in the production of reactive nitrogen species, causing apoptosis, necrosis, and autophagy due to their disturbance of the redox balance promoting cell migration [15].

Mohamed et al. have synthesized spherical mesoporous nanoparticles with diameters between 0.15 and 0.80 µm and an average pore diameter of 2.4 nm to load L-arginine, a basic amino acid model involved in several physiological processes [16]. These authors pointed out that the pH plays an important role in the degradation of the amino acid, establishing that L-arginine is in the form of zwitterion when the amino acid is adsorbed in a basic medium. However, L-arginine is positive and protonated when the adsorption takes place in an acid medium [16].

On the other hand, Gondim et al., modified the synthesis conditions of SBA-15 to obtain a porous silica with a higher pore diameter and lower channel length [17]. The adsorption capacity of these material was evaluated in human blood serum proteins such as human serum albumin and immunoglobulin G. The adsorption studies revealed that the highest adsorption values were obtained close to the isoelectric point (pI). In addition, the adsorption studies reported that these materials could be appropriated for the purification of human blood serum proteins since immunoglobulin G is much higher than that obtained for human serum albumin [17].

Martínez-Erro et al., also prepared mesoporous silica nanoparticles for delivery by structure-directing agents based on the kidney-protector drug cilastatin and using lipidic derivatives of cilastatin to direct the formation of mesoporous silica nanoparticles [18]. The releasing studies reported a progressive and slow drug release for several days, which can reduce the kidney toxicity associated with chemotherapy [18].

In the final study of this Special Issue, Ryl and Owczarz used polysaccharide matrices via thermo-induced sol–gel phase transition as drug carriers and minimally invasiveness scaffolds in tissue engineering [19]. These authors observed that the shear field formed along the injection affects to the conformation of polymer molecules and its gelation. In addition, these authors also indicated that use of low shear rates with respect to injection can accelerate the gelation, while increases in shear rates extend the gelation time; applying the highest shear rates may significantly slow down (hydroxypropyl cellulose) or accelerate gelation (chitosan). Thus, the use of thin needles without preliminary tests may lead to an extension of the gelation time and the spilling of the polymeric being carried before gelation can occur [19].
